# Family engagement in the care of infectious patients in intensive care units: A hybrid concept analysis

**DOI:** 10.1002/nop2.2117

**Published:** 2024-03-01

**Authors:** Mohsen Soleimani, Ali Fakhr‐Movahedi, Sajad Yarahmadi

**Affiliations:** ^1^ Nursing Care Research Center, School of Nursing and Midwifery Semnan University of Medical Sciences Semnan Iran; ^2^ Social Determinants of Health Research Center, School of Nursing and Midwifery Lorestan University of Medical Sciences Khorramabad Iran; ^3^ Student Research Committee Semnan University of Medical Sciences Semnan Iran

**Keywords:** concept analysis, family engagement, infectious patients, intensive care unit

## Abstract

**Aim:**

This study aims to define and investigate characteristics, antecedents, and consequences of the concept of family engagement in caring for patients with infectious diseases hospitalised in intensive care units.

**Design:**

This is a three‐phase hybrid model study (theoretical, fieldwork, and analytical phase).

**Methods:**

The York University Guidelines were used in the theoretical phase, and ultimately, 16 pieces of literature related to the subject under study from 2011 to 2021 were reviewed. The content analysis was used for fieldwork phases; eight participants were interviewed. Then, the theoretical and fieldwork findings were compared, integrated, and analysed.

**Results:**

This concept has characteristics such as; awareness, belief, perception, and willingness of the nurse to engage the family; a sense of responsibility, willingness, and sacrifice of the family; the physical or virtual presence of the family; triangular interaction between the nurse, patient, and family; perception and identifying the goals; education and information transfer; team collaboration; delegation of responsibility to the family; decision making; and protection of the family. Antecedents include the availability of infrastructure; patient, family, and nurse conditions; and the quality implementation of engagement. The consequences include positive consequences related to the patient, family, nursing, and society, as well as some negative consequences. This study provided a comprehensive perception of family engagement in the care of patients with infectious diseases in intensive care units and defined it more clearly, showing its characteristics, antecedents, and consequences.

**Patient or Public Contribution:**

Eight participants were interviewed, including five nurses, two family caregivers, and one patient.

## 1 INTRODUCTION

Intensive care units (ICUs) often have strict visiting policies for families due to concerns such as infection transmission, disruption of care, unintentional stress on the patient, and family fatigue and safety (Dragoi et al., 2022). Over the past two decades, ICUs have made significant progress towards family‐centered care and engaging families in patient care, with ample evidence demonstrating the usefulness and effectiveness of family‐centered care approaches in ICU settings. However, the emergence of the COVID‐19 pandemic in 2020 raised concerns about the implementation of family engagement approaches, as infection prevention was a fundamental principle that required restrictions or prohibitions on the presence of family members. These restrictions were initially significant due to insufficient knowledge and limited hospital capacity, given the high prevalence of COVID‐19 (Hart & Taylor, 2021). The limitations imposed on family presence in the ICU challenged the provision of family engagement (Hart et al., 2020). In addition, with the prolonged duration of the pandemic, various consequences and effects such as grief, emotional distress experienced by patients, families, and staff, communication and decision‐making barriers, disparities, and poor clinical outcomes emerged (Moore et al., 2020; Sutherland et al., 2020; Voo et al., 2020; Yardley & Rolph, 2020). Given these restrictions' serious and long‐term effects on patients, family members, healthcare workers, and communities, these limitations were no longer justifiable, and reviewing these policies became vital (Hart & Taylor, 2021).

Before the COVID‐19 era, campaigns aimed at increasing patient and family engagement in changing care policies and outcomes had effective results; some centres welcomed families not only as visitors but also as partners in care and recognised their significant role in improving the quality, safety, and clinical outcomes (Dokken & Ahmann, 2020b; Dokken et al., 2015). The Society of Critical Care Medicine recommends that during epidemics, increasing family communication and interaction should improve family‐centered care, and strategies to increase patient and family engagement should also be considered (Papadimos et al., 2018). The COVID‐19 pandemic experience has shown various reasons for family engagement during pandemics (Aboumatar, 2020). Therefore, increasing family capacity to achieve desirable outcomes in treating and caring for COVID‐19 patients has become a priority for healthcare facilities (Aboumatar, 2020). Family engagement includes family members in a joint team of the patient, and healthcare workers, a critical component of quality care (Davidson et al., 2017). During the COVID‐19 pandemic, some hospitals have reduced these limitations despite restrictions on family presence at the patient's bedside. Furthermore, some hospitals have provided conditions for Virtual visits by patients' families (Dokken & Ahmann, 2020a).

During the COVID‐19 pandemic, the role of family caregivers has become more critical than ever before (Garg et al., 2020). Patients often had greater trust in their family caregivers for choosing and receiving healthcare services. Family caregivers were regularly involved in the progress and improvement of the patient's condition and engaged in the care process, particularly in decision‐making. Upon discharge from the hospital, many patients relied on their family caregivers to help them with medication adherence, medical equipment procurement, and following post‐discharge instructions, and this required special coordination between the healthcare team and the family caregivers during hospitalisation (Aboumatar, 2020).

Family readiness could lead to better adherence to treatment plans, readmission, isolation, and decreased virus transmission within families and communities. Therefore, family engagement during infectious disease epidemics is essential for public health and welfare (Aboumatar, 2020). Various actions and approaches to family engagement in patient care during the COVID‐19 pandemic have created a new meaning of engagement that did not necessarily involve physical presence. Therefore, a new definition of family engagement in the care of patients with infectious diseases can be introduced and needs to be further examined and analysed in more detail. This study analyzes family engagement in caring for patients with infectious diseases in the ICU.

## 2 METHODOLOGY

### 2.1 Design

**FIGURE 1:**
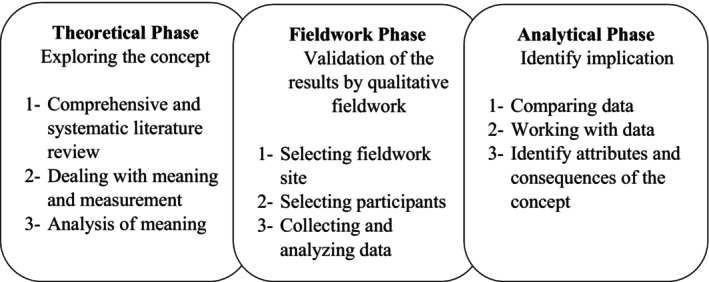
Hybrid model of concept analysis.

This study is a conceptual analysis conducted from 2021 to 2023. Since this concept has received attention since the COVID‐19 pandemic, there needed to be more literature related to this concept, and a three‐phase hybrid model (theoretical, fieldwork, and analytical phase) was used (Figure 1). The hybrid model helps to clarify, identify, analyse, and modify concepts in the early phases of theory development. This model is more commonly used in nursing (Polit & Beck, 2017). The hybrid model combines deductive and inductive approaches, aiming to identify the essential characteristics of a concept and clarify it based on participants' experiences and observations (Rahimi et al., 2021). This study can be used for concept development, concept expansion, and theoretical development in nursing to clarify ambiguities about family engagement in caring for patients with infectious diseases in the ICU.

### 2.2 Theoretical phase

The York University Guidelines were used (2009), which included selecting review questions, inclusion criteria, search strategy, study selection, data extraction, quality assessment, data synthesis, and a plan for dissemination (Cajal et al., 2020; CRD, 2009). The review questions included the definition, characteristics, antecedents, consequences of the concept, and conceptual relationship with other concepts. The keywords used for the comprehensive search included; Family Engagement, Family Participation, Family Involvement, Family‐Oriented Care, Family‐Centered Care, COVID‐19, Intensive Care Unit, and Infectious Patients. The inclusion criteria were quantitative and qualitative studies, mixed method studies, instrument development, systematic reviews, analytical articles, letters, guidelines, and dictionaries. The exclusion criteria were non‐English and non‐Persian literature and theses.

**FIGURE 2:**
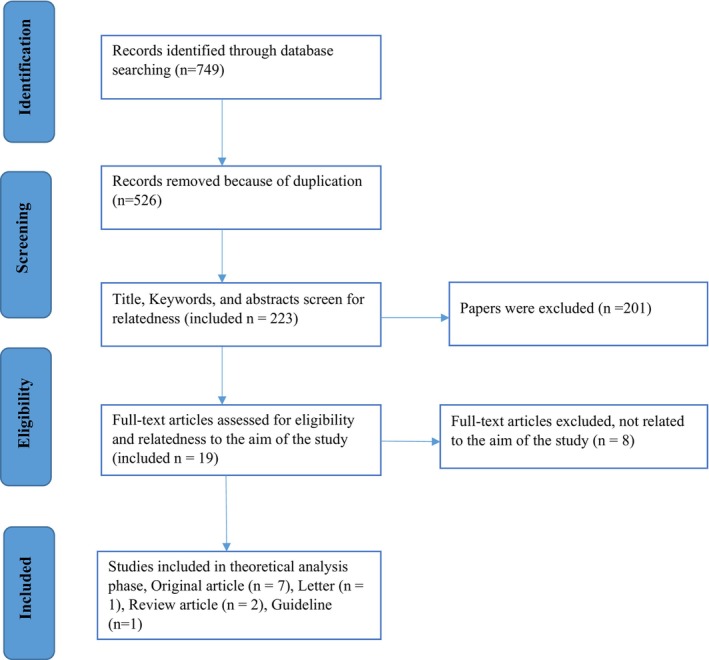
Diagram of the literature search strategy.

A systematic search was undertaken on six databases in December 2021; Pubmed, ScienceDirect, and Web of Science for English sources, and IranMedex, Scientific Information Database (SID), and Magiran for Persian (2010–2021). Online medical dictionaries such as Oxford and Webster were also used. After using search strategies in databases and removing duplicate and irrelevant studies, the remaining literature was studied, and finally, 14 articles and two related dictionaries were qualitatively analysed (Figure 2). The literature content was carefully studied, and sentences and phrases relevant to the study objectives were analysed and synthesised.

### 2.3 Fieldwork phase

This phase was conducted at the Shohadaye Ashayer Hospital, Khorramabd, Iran. In Iran, there is no distinct specialization in the field of infection prevention and control nursing. Nevertheless, each hospital typically employs an infection prevention and control nurse who has received specialized training through a dedicated course. The nursing bachelor's curriculum includes a course specifically focused on infectious diseases. Moreover, most nursing programs incorporate workshops related to infection control, along with general workshops on communication with patients and families; these communication workshops are not tailored to address the unique challenges associated with interacting with patients suffering from infectious diseases (Gorjian et al., 2023; Heidari et al., 2023).

A purposive sampling method was employed based on the study's objectives, continuing until data saturation, i.e., when the data became repetitive. The inclusion criteria comprised family caregivers, patients, and nurses with sufficient experience in family engagement in the ICU. Ten interviews were conducted involving eight participants. The desired concept was further refined and explored through semi‐structured interviews conducted in an educational setting in the field. The interview duration ranged between 60 and 90 min (mean, 67.3). Each interview commenced with the question, “Tell me about your experience with the COVID‐19 pandemic?” and proceeded with follow‐up questions such as “Can you explain further or provide an example?” or “Share your experiences.” With participants' permission, all interviews were recorded and transcribed.

The data analysis utilised the qualitative content analysis method. To thoroughly comprehend the collected information, the transcribed interviews underwent multiple close readings. Texts with similar content were categorised, and significant statements were identified as units of analysis, each assigned a specific code. Comparisons were made among various codes based on their similarities and differences to derive categories (Graneheim & Lundman, 2004). To validate codes, categories, relationships, and major themes, transcripts were revisited, and subthemes and themes were discussed among the researchers engaged in the study. A collaborative approach within the research team was adopted to enhance the study's credibility. Participants received interview transcripts and emerging categories for confirmation, and unanimous agreement was achieved with the researchers' conclusions. The data were managed and organised using MAXQDA software (Version 2020).

During the fieldwork phase, to ensure the credibility of the findings, four criteria proposed by Lincoln and Guba (1985), including credibility, transferability, dependability, and confirmability, were considered (Lincoln et al., 1985; Polit & Beck, 2017). A series of measures were taken to ensure the findings' accuracy and validity, including memo writing, field notes, peer and member checking, long‐term interaction with the environment, audit trail, and detailed descriptions of interviews.

### 2.4 Analytical phase

The findings from the previous phases were compared, integrated, and analysed.

## 3 FINDINGS

### 3.1 Theoretical phase

#### 3.1.1 Definition, characteristics, antecedents, and consequences

According to the Oxford Online Dictionary, “engagement” means the arrangement of an official task or a job related to a specific time (oxford). As stated in Webster's Dictionary, the word “engagement” has French roots, and its applications are mainly related to when someone shares a valuable asset with another person, they become committed to them. They cannot leave them (webster).

According to Webster's Dictionary, the meaning of “family” can vary for different individuals and can have different meanings. The first applications of the word family indicate a “group of individuals in service to a person.” The root of this word comes from “familia,” meaning “house,” a term that includes servants and relatives. In modern applications, the family may collectively refer to various groups of people or objects, such as chemical compounds, related languages, plants and animals, and individuals with a common lineage. In many legal contexts, family refers to “individuals related by blood, marriage, or adoption.” However, in others, this definition may be somewhat broader and include groups of individuals unrelated to these factors (webster). In general, the family can be defined into three broad categories. The first category is the structural definition, usually based on the presence of specific individuals who live together in a house. The second category is the functional definition, which focuses on families' social functions, such as child‐rearing, emotional and material support, and reproduction. The third category is typically exclusive definitions, usually based on familial identity, emotional ties, and shared past and future (Miller, 2016).

Family engagement refers to actively involving families in supporting and promoting a patient's health and influencing healthcare decisions. Engagement shifts the focus from “taking action to improve health for people” to “taking action with people.” Family engagement at every level of the healthcare system is carried out without restriction to improve health, quality, safety, and the provision of healthcare, including direct care; this is done while considering the patient's goals while preserving the family's and patient's values, respect, and dignity (Brown et al., 2015; Burns et al., 2018).

The family includes all individuals the patient wants to engage in their care, regardless of biological, legal, or unrelated relationships. If the patient is not in contact with anyone, healthcare workers will try to identify and include individuals whom the patient wants to engage in their care (Brown et al., 2015). Family engagement in ICU refers to family caregivers' presence in ICU settings and direct collaboration in aggressive interventions or patient resuscitation (McAndrew et al., 2020). Family caregivers' engagement is a critical component of high‐quality ICU care. However, the restrictions on visitation policies during the COVID‐19 pandemic could have hindered this engagement. Innovative family engagement and support models in the ICU during visitation restrictions, such as the COVID‐19 pandemic, are needed. Therefore, some studies have emphasised the need for guidelines and strategies to enhance family engagement during the COVID‐19 pandemic (Taylor, Short, et al., 2020; Taylor, Short Iii, et al., 2020). These include using appropriate personal protective equipment, individual education (Siddiqui, 2021), facilitating communication between the patient care team and family members, developing humanistic nursing, and providing emotional support to families (Taylor, Short Iii, et al., 2020). Table 1 shows the other findings of the theoretical phase.

**TABLE 1:** Different dimensions of family engagement in ICU during the epidemic of infectious diseases from the literature review.

Authors (year)	Study objective	Characteristics	Antecedents	Consequences
Abid et al. (2021)	Ways for enhancing patient and family engagement in post‐COVID conditions	Collaborative healthcare services	Increasing the use of technology for engagement Utilising monitoring policies to align with engagement strategies Equity and justice for patient and family engagement	–
Dokken and Ahmann (2020a)	Solutions for enhancing patient and family engagement	Common goal Communication and education Decision‐making	Minimising risks for families Pre‐evaluating the setting before the family presence Creating compassionate exceptions for paediatric patients, pregnant women, patients with cognitive and communication disorders, or end‐of‐life stages Supportive communication for patients whose families are unable to engagement	Quality improvement Enhancing safety Increasing post‐discharge education and follow‐up
Skoog (2016) Khalaila (2014)	Investigating the level of anxiety in families of hospitalised patients in the cardiac surgery ICU following a family engagement facilitation program	Identifying and estimating information needs Training for clinical care activities Training on how to engage and assess their own needs	Assisting families in understanding the ICU setting Providing family support	–
McAndrew et al. (2020)	Obtaining feedback from families of patients hospitalised in the ICU to improve quality through family engagement facilitated by nurses	Family interaction with interdisciplinary teams. Sharing information. Effective communication	Guiding families in the ICU setting	Improving the quality of care
Kruser et al. (2019)	Describing decision‐making patterns, how and when hospitalised patients in the ICU and their families engage in treatment decisions	–	Engagement in the care of chronic patients Engagement in the care of patients at risk of death.	Achieving physiological goals
Naef et al. (2021)	Describing nurses' understanding and performance of family engagement in ICU from a global perspective	Nurse‐family communication. Information transfer to families Team collaboration. Nurses' different perceptions. Families' different perceptions	The resources and structure of the ICU The culture of ICU	–
Hetland et al. (2018)	Describing the approaches of ICU nurses to enhance family engagement in direct patient care	Determining participatory care. Determining how to implement care	Accurate assessment. Encouragement of families by nurses. Effective factors, including; family factors, patient factors, nurse factors, professional practice environment, unit, and organisational resources	–
Olding et al. (2016)	Assessing the scope of patient and family engagement	Presence Support Engagement in communication Engagement in clinical care Engagement in decision‐making	–	–
Black et al. (2011)	The impact of family engagement in psychological care on reducing patient delirium and improving psychological recovery following critical illness	–	–	Improving the psychological well‐being of the family
Brown et al. (2015)	Definition of patient and family engagement	Active interaction between families and healthcare professionals Empowerment in acquiring knowledge and skills Information sharing Decision‐making	Respecting the dignity and status of the family and patient in planning Compassionate behaviour towards the family Encouragement of families Family support	Quality Safety Health
Taylor, Short, et al. (2020)	Rapid assessment of facilitators and barriers in designing and implementing executive strategies to support a new program using medical students to facilitate family‐centered care in COVID‐19 ICU patients	–	–	Increase communication among family members, patients, and the healthcare team Improve family knowledge about communication during patient hospitalisation Increase the use of humanisation strategies. Increase the duration of family presence at the bedside Increase the satisfaction of family, patients, and staff Increase family engagement in decision‐making Reduce stress disorders in families, including anxiety and post‐traumatic stress disorder Reduce stress disorders among patients Alignment of patient care with defined goals

#### 3.1.2 Conceptual relationship with other concepts

**FIGURE 3:**
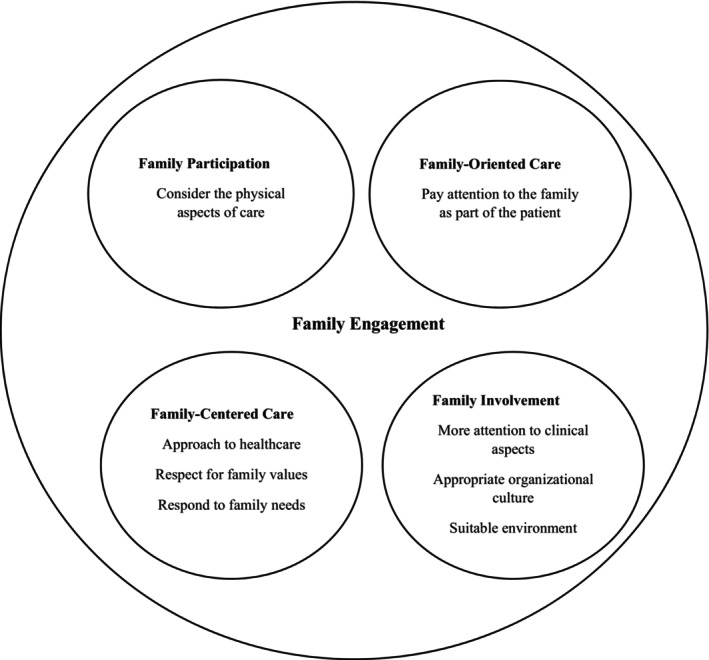
The relationship of the Family Engagement concept with other concepts.

Family engagement is also related to other terms, including family‐centered care, family‐oriented care, family involvement, and family participation. However, these concepts are distinct from each other. Family engagement can encompass a broader range of these concepts. Family‐centered care is an approach to healthcare that respects and responds to the values and needs of family members (Davidson et al., 2017). In this regard, family‐centered care is one of the possible outcomes of family engagement, where engagement may be considered a mechanism for achieving family‐centered care (Burns et al., 2018). Family involvement has focused more on the clinical aspects of involvement. However, organisational culture and a suitable work environment are necessary for achieving family involvement (Hetland et al., 2018), which is also required for family engagement (Dokken & Ahmann, 2020a). In the analysis of family participation in the ICU, emphasis has been placed more on the physical aspects of care (Lee & Craft‐Rosenberg, 2002). Family‐oriented care considers the family an integral part of the patient that requires attention, and the family also cares for the patient (Askari et al., 2013) (Figure 3).

### 3.2 Fieldwork phase

In this phase, eight participants were interviewed, including five nurses (three females and two males), two family caregivers (one female and one male), and one patient (male). Participants' mean (SD) age was 37.95 (6.52) years. One participant had a master's degree, five had a bachelor's degree, and two had a diploma.

#### 3.2.1 Characteristics

One of the essential characteristics of engagement in care is interaction and communication. This interaction involves the patient, family, nurse, or healthcare team; family engagement can happen with these three components. Of course, having a suitable setting for this interaction, either in‐person or remotely, is necessary. The results show that the nurse's belief and perception of family engagement are essential, and the family's engagement in caring for an infectious disease patient hospitalised in the ICU during the pandemic starts with the nurse's engagement with the family. However, the family's willingness and perception are also essential. However, there are also limitations to engaging the family in the care process that cannot be optional for the nurse. Participant 1 stated, “The head nurse, shift supervisor, or hospital supervisor can restrict the family's engagement for us. At first, the hospital management did not even allow visits.” The presence of the family is necessary for family engagement. The family can engage in care either physically or virtually. The family's protection and personal protective equipment are essential during the family's presence. One of the critical requirements for engaging the family in care is to set a common goal for involving the family in a specific matter or issue, and mutual perception of this goal is necessary for the nurse, family, and patient. The family must be aware of the patient's condition to be engaged.

In this process, nurses delegate some of their responsibilities to the family. The nurse determines the extent of family engagement. Participant 2 said, “For example, we don't engage the family in everything; we delegate the tasks that we have in mind to the family; for example, we don't ask for their help in giving medication.” One of the essential characteristics of this concept is the family's engagement in decision‐making in the care process. Participant 3 said, “We didn't have Entera Meal in the ward; the patient's daughter was in the ward; I told her we don't have gavage feeding powder in the hospital; what should I do? She said, now I'll make banana milk for him, and for his dinner, I'll bring some muscle soup for dinner; I won't give him the hospital's mixed soup, the last time we gave it to him, it upset his digestion.” One of the prerequisites for this concept is the sacrifice and selflessness of the family members who, knowing that they may become infected, are present at the patient's bedside and take care of the patient. Participant 4 said, “When I saw that I was alone and unmarried, my life was over; I decided to go over my nephew's head myself; I thought that if it happened to me, maybe no one would get hurt. But if my brother went to his son's bedside, there might be a problem for him, then what would happen to his wife and child? I have passed myself.” Awareness of essential health and personal protection principles is necessary for family engagement, and before entering the field, the family must be aware of these issues or be trained. Participant 1 said, “We gave them masks, gloves, and gowns, and we also gave them some preventive education, then we brought them into the ward.”

#### 3.2.2 Antecedents

When the family or patient requests engagement, it can be an antecedent for family engagement. Of course, allowing the nurse's permission is also necessary here. Family engagement increases when the patient's condition is uncertain, the advance notice of death is high, there is fear of the patient's death, and the patient experiences sadness and loneliness. Participant 5 said, “Our patient was intubated and crying; we used to ask his son to come next to him and do some things for him.” Family engagement increases when the care process is prolonged, and there are favourable clinical outcomes. Participant 3 said, “The patient's hospitalisation period had become long. How could he be kept away from his family, especially since some cases such as bathing, facial correction, and other tasks had become necessary over time, the family had to be present.” When the nurse's workload increases, the nurse tries to engage the family in the care process. Participant 6 said, “Well, at that time, our workload was very high; we asked the families for help to reduce our workload.” One of the prerequisites is a suitable space for family engagement. Family engagement increases if suitable space or family comfort and convenience are provided. Another antecedent is the shape of family relationships, which can influence family engagement. Participant 7 said, “The son of my roommate, who was in the next bed, said, we got a wife for our father to take care of him, now he doesn't come to the hospital and tells me that his children are not near his bed, I pray my father is released from here, we will divorce his wife.” Additionally, providing emotional and psychological support to the patient is one of the antecedents.

#### 3.2.3 Consequences

Family engagement has multiple positive and negative consequences. Positives such as patient relaxation and improved patient physical health. Participant 2 stated, “The family caregivers bring them strengthening foods or herbal teas.” Other consequences include addressing care deficiencies that may arise from nursing neglect. Participant 8 stated, “The nurse was busy and forgot to change the position of my patient. I turned to the maid and asked her to help me do this together so the wound wouldn't be bedridden. Also, the physiotherapist often did not come, or when he did, he spent little time with the patients, and I would give my patient some stretching exercises.” With family engagement, the family can perform neglected care tasks that nobody else does or has time for. Another consequence is the reduction of nurse responsibilities and subsequently reducing their workload.

However, educating families can increase the workload for nurses. One necessary consequence is the improvement of patient safety. Participant 5 stated, “The family of the patient who is present is somewhat comforting because they are with the patient, the patient does not disconnect the connectors, the possibility of falling is reduced, or they are also monitoring the patient.” Participant 2 stated, “Some families would come for their patients and give them massages. Given that they were at risk of blood clots, their families came and massaged their legs and arms to prevent DVT.” Another consequence is the acceleration and facilitation of care and treatment. When healthcare workers have limited resources to perform specific interventions, they turn to family caregivers for help, such as when some medications are rare. Family presence in the ICU can also provide family caregivers with learning and education opportunities regarding disease awareness, health promotion, and disease prevention. It can also help spread this knowledge to the community. Participant 4 stated, “I learned a lot there. One of the nurses taught me how to wash my hands better or to be careful not to touch my face. After my patient's discharge, I often taught those things to relatives who had COVID.”

Additionally, by being present in the field, families can learn home care techniques through deeper interactions and improve their care skills for home care. Another necessary consequence is improving three‐way communication and the sense of family solidarity. Participant 7 stated, “Whenever I remember how much my brother helped me at that time, my relationship with him gets much better.” Participant 2 stated, “When I brought his wife into the ward, our patient's relationship with me improved significantly.” With the development of communication, nurses can expand their counselling role. Participant 1 stated, “Many families came and asked me questions; for example, one of the patients' wives who had women's problems came and asked me questions.”

Additionally, by being present in the field, families can learn home care techniques through deeper interactions and improve their care skills for home care. Another necessary consequence is improving three‐way communication and the sense of family solidarity. Participant 7 stated, “Whenever I remember how much my brother helped me at that time, my relationship with him gets much better.” Participant 2 stated, “When I brought his wife into the ward, our patient's relationship with me improved significantly.” With the development of communication, nurses can expand their counselling role. Participant 1 stated, “Many families came and asked me questions; for example, one of the patients' wives who had women's problems came and asked me questions.”

Other positive consequences are an increase in trust in healthcare workers, family peace, and an improvement in the perception of nurses in society. Participant 6 said, “When families would come and see that we don't even have time to sit and take a short break, they would come and thank us; this happened often. Alternatively, outside the hospital, I often heard them praising us nurses because they had seen it with their own eyes.” Another consequence is increased patient comfort following patient care with patience and care from the family. Developing a sense of empathy and social participation in a time when society needs help is another positive consequence. Participant 3 said, “One of the family caregivers would come and help not only their patient but also other patients, saying, ‘I want to help in this situation so that, God willing, we can get out of this crisis.’” The presence of families in the ICU environment can change the atmosphere and eliminate the feeling of being mechanical. With the presence and engagement of families, it is possible to quickly obtain consent for any intervention, facilitating the decision‐making process and reducing the legal burden of interventions. Also, family engagement can allow nurses to view the patient and family as a whole, as family‐patient relationships cannot be ignored, enhancing holistic care.

The negative consequences are the Nurses feeling monitored, the risk to the family's health, and the spread of disease in society. Participant 3 said, “When we had to intubate the patient, their family was screaming and yelling, saying that we had killed their loved one.” Inadequate family comfort in ICU environments is also another negative consequence. Participant 8 said, “There were times when there wasn't even a chair to sit on.”

### 3.3 Analytical phase

The final analysis indicates that the concept encompasses characteristics such as the nurse's awareness, belief, perception, and willingness to engage the family; the family's sense of responsibility and willingness to sacrifice; the family's physical or virtual presence; the triangular interaction involving the nurse, patient, and family; goal perception and identification; education and information transfer; collaboration within the team; delegation of responsibilities to the family; decision‐making; and protection of the family. Antecedents involve factors such as infrastructure availability, patient, family, and nurse conditions, and the quality of engagement implementation. Consequences encompass both positive outcomes for the patient, family, nursing, and society, as well as negative consequences. The findings of the final analytical phase are presented in Table 2.

**TABLE 2:** The findings of the final analytical phase.

Characteristics	Awareness, belief, perception, and willingness of the nurse to engage the family Sense of family responsibility, family willingness, and sacrifice Physical or virtual presence of the family Triangular interaction between the nurse, patient, and family Understanding and identifying the goals Education and information transfer Team collaboration Delegation of responsibility to the family Decision making Protection of the family
Antecedents	Availability of infrastructure: Alignment of system policies and ICU culture; appropriate physical setting for the comfort and well‐being of the family; remote communication technology to prevent transmission of infection Conditions of the patient, family, and nurse: the feeling of homesickness or loneliness in the patient and family; fear of the patient's death; high death prognosis; increasing nurses' workload Quality implementation of engagement: maintaining justice and equality between patients and families; complying with compassionate exceptions; encouraging and persuading the family to engage; patient and family request for engagement; support, guidance, respect, preservation, and kind behaviour with the family; helping the family to identify and understand the setting; accurate assessment of engagement by the nurse
Consequences	Positive consequences related to the patient and family: improvement of quality, safety, satisfaction, comfort, education and psychological and psychological support; improvement of follow‐ups after discharge and faster attainment of health; increase of knowledge and skills, family well‐being, improvement of interactions and communication, and a sense of empathy and solidarity, changing the mechanical setting of the ICU The positive consequences of nursing: the development of humane nursing, holistic nursing, and the advisory role of nurses, increasing satisfaction and trust in nurses, eliminating care deficiencies, reducing responsibilities and workload, and then reducing the legal burden of nursing, accelerating and facilitating care, and treatment, improving the perception of nurses in society Positive consequences related to the community: promotion of health and disease prevention, development of general education and improvement of community and caregiving skills, development of collective empathy, and social participation Negative consequences: family caregivers being at risk and contracting the infection; spread of the disease in the community; Insufficient family comfort, overcrowding in times of crisis; and feeling of being under the supervision of nurses by the family caregivers

## 4 DISCUSSION

In the current study, our goal was to delineate and explore the characteristics, antecedents, and consequences of the concept of family engagement in caring for patients hospitalised with infections in intensive care units. Employing a hybrid concept analysis, we aimed to furnish a comprehensive understanding of the intricate nature of family engagement within the critical care setting during infectious episodes. The study findings not only defined the concept of family engagement in the care of patients with infectious diseases in the ICU but also illuminated its associations with analogous concepts. Additionally, it identified the specific characteristics, antecedents, and consequences associated with this concept.

During an epidemic, it is necessary to adjust the goals according to the rapidly changing clinical culture. A study has indicated that family engagement goals in caring for COVID‐19 patients include respecting the role of family as care partners, collaboration between family caregivers and healthcare teams, and maintaining family cohesion (Hart et al., 2020). Family engagement primarily relies on the physical presence of family caregivers beside the patient's bed to promote trust, communication, participation in care, and joint decision‐making. During pandemics, family presence should also be supported and encouraged through virtual means to achieve family engagement goals (Hart & Taylor, 2021).

Although cultural differences exist worldwide, the methods and challenges related to family engagement and collaboration in ICUs are generally familiar. Existing studies emphasize the importance of addressing communication, cultural, and structural aspects in research to strengthen family engagement in the ICU (Hamilton et al., 2020; Hetland et al., 2017, 2018; Kiwanuka et al., 2019; Østergaard et al., 2020; Scott et al., 2019; van Mol et al., 2017). In a qualitative study that examined the perspectives of nurses from five countries on family engagement in the ICU, it was found that family engagement is related to; the power of nurses in establishing relationships and interacting with the patient's family, as well as their ability to convey information; individual and performance differences among nurses, as well as differences among patients and their families; the context of the ICU, which is related to the culture of the ICU, teamwork, and the structure and resources available in the ICU (Naef et al., 2021).

Some studies have suggested solutions for engaging the families of COVID‐19 patients in the ICU. These include receiving healthcare services from family members, maintaining communication through various means, and involving families in decision‐making (Goldfarb et al., 2020). Life‐sustaining care and treatment in the shadow of the preferences and values of hospitalised patients in ICU pose multiple challenges. Many patients prefer to avoid intensive medical treatments, while others prioritize quality of life over life prolongation in the end‐of‐life stages (Kruser et al., 2019). Shared decision‐making has been recommended to overcome these challenges (Davidson et al., 2017; Kon et al., 2016), which requires family engagement in decision‐making and consultation regarding the patient's goals and preferences. Shared decision‐making is recommended for treatment decisions involving uncertainty, high‐risk, or potentially unacceptable patient outcomes (Detsky et al., 2017; Kruser et al., 2019). During patient hospitalisation in the ICU, multiple treatment decisions are made sequentially. These decisions are sometimes made urgently or daily, and patient preferences may influence the outcomes. However, the timing and manner of patient and family engagement in these multiple treatment decisions still need to be fully understood (Bruce et al., 2013; Kruser et al., 2017). In addition, family engagement in decision‐making is crucial for critically ill patients with chronic conditions who remain hospitalised in the ICU for months (Kahn, 2015).

Hospitalisation of a family member in the ICU can be stressful for other family members, with stress levels reported up to 80%. It has also been reported that up to three months after discharge, the level of anxiety in family members remains high. Family caregivers need to understand the ICU setting to discover their caregiving role, and failure to do so may result in psychological problems such as anxiety in the family. Through some actions, the nurse helps to increase the sense of family engagement. These actions include: helping the family understand the ICU environment, identifying and meeting the information needs of family caregivers, teaching them how to visit and assess their own needs, providing family support, and providing activities for clinical care at the bedside. These activities are essential for family satisfaction, meeting family needs, and maintaining communication in the ICU, which can lead to reduced anxiety and increased satisfaction (Fumis et al., 2015; Khalaila, 2014; Skoog et al., 2016). Skoog et al.'s study (2016) demonstrated that using nursing interventions to increase family engagement in caring for patients hospitalised in the cardiac surgery ICU reduced family anxiety levels (Skoog et al., 2016).

Family engagement in the care of ICU patients was recommended in healthcare systems before COVID. It has been confirmed that the presence and engagement of the family positively affect the patient's recovery. The patient's family's intrinsic motivation to accompany and support the patient can be an essential resource for family‐centered care (Burns et al., 2018). However, the COVID‐19 pandemic has caused experiences of unknown fear, uncertainty, financial burden, anxiety, depression, role change, and increased responsibilities to family members. These cases could affect the family's mental health and their reconciliation, and when the family cannot visit the patient, these conditions become even direr. Facilitating communication processes for family participation is needed not only in the decision‐making process but also to improve the mental state of families. It is necessary to engage the family and, at the same time, use protective protocols to prevent infection to prevent psychological damage to the patient and their family (Saghafi et al., 2021).

A qualitative content analysis study identified five categories of facilitators related to family engagement of patients admitted to the ICU. These categories include family‐related factors, such as the engagement and permanent presence of families; patient‐related factors, such as the patient's positive response to family care, which reduces fear and anxiety; nurse‐related factors, such as recognition of the benefits of family engagement, previous experience, positive personal philosophy, strong communication skills, and motivation to overcome system barriers; environmental factors such as adequate staffing that allows a focus on family care, consistency in providers, inter‐professional support, family engagement inter‐professional courses, and supportive nurse leaders; and organisational resources such as unrestricted visiting policies, the existence of frameworks, guidelines, policies, and protocols, private rooms, and the physical design of the ICU (Hetland et al., 2018).

Also, barriers related to family engagement of patients admitted to the ICU are categorised into five categories. Barriers related to the family include unstable mood, low health literacy, non‐acceptance of risks or policies, lack of respect for boundaries or nurses, family conflict, family dynamics, family members who express a desire to be engaged but are not present, conflicts between nurses and families, and unrealistic expectations of nurses from the family. Obstacles related to the patient include acute critical condition, psychological problems, and the presence of lines, tubes, and equipment. Nurse‐related barriers include previous negative experiences, uncertainty about how to engage family caregivers, and discomfort with deviating from established practices. Environmental barriers include inappropriate staffing ratios, negative nursing culture, inadequate leadership and inter‐professional support, and poor communication among healthcare workers. Barriers related to organisational resources include a need for more policies and guidelines, inadequate physical space and comfort facilities for families, restrictions or incompatibility of nurses for family visits, and language or cultural barriers (Hetland et al., 2018). In line with the results of this study, McConnell and Moroney (2015) identified several factors, such as attitude towards engagement, experience, the need to imagine fulfilling one's duty, and unwillingness to perform care duties in the presence of relatives, as obstacles to nurses' willingness to engage family caregivers (McConnell & Moroney, 2015).

Family engagement in the care of these patients is not without risks (Clay & Misak, 2016; Haines et al., 2017). The results of the present study showed that one of the disadvantages of family engagement is the risk of family caregivers contracting patients with infectious diseases and accelerating their transmission to society. Virtual communication can be used as a means of preventing the transmission of infectious diseases to family caregivers; however, it may compromise patients' privacy and increase racial, social, economic, and geographic disparities for those who lack access to the internet, trust, or technological literacy (Hart et al., 2020). The results of other studies show that the disadvantages of family engagement may include psychological risks for families related to the experience of being in the ICU, as well as risks to the relationship between medical staff and patients, such as criticism of care and families feeling pressured to engagement (Haines et al., 2017).

Conducting this study had limitations, including changes in clinical culture due to the evolving nature of the disease during the COVID‐19 pandemic. This issue could have affected the dimensions of the concept. However, efforts were made to focus on the fixed and central aspects of the concept.

As a pathway for future research, we propose investigations into long‐term impacts, cross‐cultural dynamics, technological interventions, and the effectiveness of educational programs to enhance family involvement in critical care settings. These avenues of inquiry aim to continually refine healthcare practices, optimising patient and family‐centered care during infectious episodes in intensive care units.

## 5 CONCLUSION

This study provides a comprehensive perception of the concept of family engagement in patients hospitalised in ICU during the epidemic of infectious diseases and defines it more clearly. The findings show that this concept has characteristics such as the awareness, belief, perception, and willingness of the nurse to engage the family; the sense of family responsibility, willingness, and sacrifice; the physical or virtual presence of the family; triangular interaction between the nurse, patient, and family; perception and identification of goals; education and information transfer; team collaboration; delegation of responsibility to the family; decision‐making; and protection of the family. Antecedents include the availability of infrastructure, patient, family, and nurse conditions, as well as the quality of implementation of engagement. The consequences include positive consequences related to the patient, family, nursing, and society, as well as negative consequences.

## AUTHOR CONTRIBUTIONS

Mohsen Soleimani: Conceptualisation, Methodology, Investigation, Data Curation; Data Analysis; Writing – Original Draft. Ali Fakhr‐Movahedi: Conceptualisation, Methodology, Writing – Original Draft. Sajad Yarahmadi: Conceptualisation, Methodology, Investigation, Data Curation; Data Analysis; Writing – Original Draft.

## FUNDING INFORMATION

There was no specific grant for this research from government, private, or nonprofit funding organisations.

## CONFLICT OF INTEREST STATEMENT

The authors affirm that no financial or commercial relationships might be seen as having a conflict of interest.

## ETHICS STATEMENT

The study's purpose and procedures were clearly explained to all participants, and their informed consent was obtained. Participants were assured that their voices would be recorded, transcribed, and kept confidential. This study was approved by the Ethics Committee of Semnan, University of Medical Sciences, Semnan, Iran (IR.SEMUMS.REC.1400.161).

## Data Availability

The data that support the findings of this study are available from the corresponding author upon reasonable request.
